# Quality of life assessment in chronic wound patients using the Wound-QoL and FLQA-Wk instruments

**DOI:** 10.17533/udea.iee.v38n3e11

**Published:** 2020-11-11

**Authors:** Tatiele Naiara Vogt, Francisco José Koller, Pamella Naiana Dias Santos, Bruna Eloise Lenhani, Paulo Ricardo Bittencourt Guimarães, Luciana Puchalski Kalinke

**Affiliations:** 1 Nurse, Master. Email: tatielevogt@hotmail.com tatielevogt@hotmail.com; 2 Nurse, PhD student. Teacher. Email: enfkoller@yahoo.com.br enfkoller@yahoo.com.br; 3 Nurse, Master. Email: pamella.nds@gmail.com pamella.nds@gmail.com; 4 Nurse, PhD. Email: bru_lenhani@hotmail.com. bru_lenhani@hotmail.com; 5 Statistician, PhD. Adjunct Professor III. Email: guimaraes.prb@gmail.com. guimaraes.prb@gmail.com; 6 Nurse, PhD. Adjunct Professor III. Email: lucianakalinke@yahoo.com.br lucianakalinke@yahoo.com.br; 7 Joinville City Hall. Joinville, Santa Catarina - Brazil. Prefeitura Municipal de Joinville Joinville City Hall Brazil; 8 Santa Cruz Integrated Faculties. Curitiba, Paraná - Brazil. Faculdade Santa Cruz Santa Cruz Integrated Faculties Curitiba Brazil; 9 Afonso Pena Emergency Care Unit. Curitiba, Paraná - Brazil. Afonso Pena Emergency Care Unit Curitiba Brazil; 10 Hospital Erasto Gaertner, Curitiba, Paraná - Brazil. Hospital Erasto Gaertner Curitiba Brazil; 11 Federal University of Paraná, Curitiba, Paraná - Brazil Universidade Federal do Paraná Federal University of Paraná Curitiba Brazil

**Keywords:** wounds and injuries, quality of life, questionnaires, nursing care, leg ulcer, diabetic foot, heridas y traumatismos, calidad de vida, cuestionarios, atención de enfermería, úlcera de la pierna, pie diabético, ferimentos e lesões, qualidade de vida, questionários, cuidados de enfermagem, úlcera da perna, pé diabético

## Abstract

**Objective.:**

To evaluate changes in the quality of life of patients with chronic wounds.

**Methods.:**

Quantitative research with a cross-sectional design performed with 100 patients with chronic wounds from a university hospital and a Basic Health Unit in southern Brazil. The mean values of the domains of the instruments Wound Quality of Life (Wound-QoL) and Freiburg Life Quality Assessment Wound were compared with sociodemographic variables of age, sex and education.

**Results.:**

The average age of the participants was 60.98 years old; 41% (n = 41) had diabetic ulcers and 83% (n = 83) treated the wounds for more than 24 months. The total quality of life value was below the mean with 37.50/100 with (Wound-QoL) and 44.20/100 with (FLQA-Wk). The variables of gender, and educational level were not correlated with either of the two instruments used to assess the quality of life. The age variable was significantly correlated with the satisfaction item of the FLQA-Wk.

**Conclusion.:**

The quality of life of patients with chronic wounds was considered poor. The age variable was correlated with the satisfaction domain, showing that the older the age, the lower the satisfaction. The use of instruments to evaluate the quality of life of patients with chronic wounds may help an effective treatment plan.

## Resumen

**Objetivo.:**

Evaluar cambios en la calidad de vida de pacientes con heridas crónicas.

**Métodos.:**

Investigación cuantitativa con diseño transversal, realizada en 100 pacientes con heridas crónicas de un hospital universitario y una Unidad Básica de Salud en el sur de Brasil. Se compararon las medias de los dominios de los instrumentos *Wound Quality of Life (Wound-Qol) y Freiburg Life Quality Assessment Wound* - Versión heridas (FLQA-Wk), con variables sociodemográficas edad, sexo y educación.

**Resultados.:**

La edad promedio fue de 60.98 años; 51 pacientes eran hombres, 41 tenían úlceras diabéticas y 83 trataron las heridas durante más de 24 meses. La calidad de vida total tuvo valores por debajo de la media 37.50 / 100 con Wound-Qol y 44.20 / 100 con FLQA-Wk. Las variables sexo, nivel educativo no se correlacionaron con ninguno de los dos instrumentos utilizados para evaluar la calidad de vida. La variable edad se correlacionó significativamente con el ítem de satisfacción de la FLQA-Wk.

**Conclusión.:**

La calidad de vida de los pacientes con heridas crónicas se consideró mala. La variable edad se correlacionó con el dominio de satisfacción, lo que muestra que a mayor edad, menor satisfacción. El uso de instrumentos para evaluar la calidad de vida de los pacientes con heridas crónicas puede ayudar en la realización de un plan terapéutico eficaz.

## Resumo

**Objetivo.:**

Avaliar as alterações na qualidade de vida de pacientes com feridas crônicas.

**Métodos.:**

Pesquisa quantitativa com delineamento transversal, realizada em 100 pacientes com feridas crônicas de um hospital universitário e de uma Unidade Básica de Saúde no sul do Brasil, foram comparadas as médias dos domínios dos instrumentos Wound Quality of Life (Wound-Qol) e Freiburg Life Quality Assessment Wound - Versão Feridas (FLQA-Wk), com as variáveis sócio-demográficas idade, sexo e escolaridade.

**Resultados.:**

A média de idade foi de 60.98 anos; 51 pacientes eram do sexo masculino, 41 tinham úlceras diabéticas e 83 tratavam as feridas por mais de 24 meses. A qualidade de vida total teve valores abaixo da média, 37.50/100 com o Wound-Qol e 44.20/100 com o FLQA-Wk. As variáveis sexo, escolaridade não se correlacionaram com nenhum dos dois instrumentos usados ​​para avaliar a qualidade de vida. A variável idade foi correlacionada significativamente com o item satisfação da FLQA-Wk.

**Conclusão.:**

A qualidade de vida dos pacientes com feridas crônicas foi considerada ruim. A variável idade se correlacionou com o domínio satisfação, denotando que quanto maior idade, menor é a satisfação. A utilização de instrumentos para avaliação da qualidade de vida de pacientes com feridas crônicas poderá auxiliar na realização de plano terapêutico efetivo.

## Introduction

Chronic wounds (CW) affect approximately 1 to 2% of the world population.(1) In Brazil, there is a high number of patients with wounds. A review on the theme carried out in the country from 2003 to 2014 showed the prevalence of leg ulcers (40%) followed by diabetic ulcers (33%).(2) There has also been an increasing number of patients with CW, and costs follow the same trend, causing an impact on family budgets and on health systems. In 2014, a total of 22,244 patients diagnosed with diabetes mellitus with procedures related to diabetic foot were hospitalized in Brazil, with a total cost of $ 27.7 and $ 333.5 million dollars for inpatient and outpatient care, respectively (1US dollar = R $ 3.88 reais).(3)

There has been an increase in the appearance of CW and on the financial impact caused by them, and on the other hand, a decrease in the quality of life (QoL) of affected patients. Physical, social and emotional damage such as decreased mobility, pain and discomfort, unpleasant odor and insomnia, tend to limit the performance of daily activities.(4) Social isolation, frustration and other psychological reactions, such as anxiety and depression, are also factors that add to the physical aspects, and cause an impact on the lives of patients.(5) Throughout the therapeutic course, QoL should be measured as an assessment of the treatment and of the evolution of the disease, aiming to minimize the effects that CW cause in the lives of patients.

Among the various definitions of the construct “Quality of life”, the one of the World Health Organization Quality of Life Group was used in this study: “an individual’s perception of their position in life in the context of the culture and value systems in which they live and in relation to their goals, expectations, standards and concerns”.^(6:1)^ This definition aims to express the extent and multidimensionality of the construct, covering health, social and economic aspects. However, exclusive and reliable tools are necessary for the assessment to encompass the multidimensionality of QoL. Thus, there is a need for instruments to help professionals to obtain accurate information about the patients’ conditions so as to develop assertive plans aimed at improving QoL.(7)

Chronic wound patients tend to suffer changes in QoL related either to the physical limitations that the wound may cause or to restrictions in the access to treatment. Age, low schooling, income, and psychological problems are essential variables to be considered when the therapeutic itinerary is planned, due to the limitations that they can cause. The number of elderly people in the Brazilian population is on an upward curve, reflecting a greater incidence of comorbidities as age increases. Schooling, for example, is considered an important factor to be evaluated, because it promotes the patients’ understanding of their disease, facilitating treatment and adherence.(8)

In the context of assessing the QoL of patients with CW, the objective of this research emerges: To assess changes in the QoL of patients with chronic wounds and compare the average values of the domains of the Wound Quality of Life (Wound-QoL) and the Freiburg Life Quality Assessment Wound - Wound Version (FLQA-Wk), with the sociodemographic variables age, sex and education.

## Methods

This is a quantitative study with a cross-sectional design carried out in two outpatient clinics, one of a large university hospital (UH) and another of a Basic Health Unit (BHU), characterized as an institution that provides primary care to the population. Both are funded by the government and specialized in wound care, located in a capital in southern Brazil. Data collection was performed in a single moment between December 2017 and April 2018, using three instruments: the first developed by the researchers for collection of information on sociodemographic and clinical characteristics, and the other two were the instruments that measure QoL, the Wound-QoL and FLQA-Wk.

Among the specific instruments used to assess the QoL of patients with CW, the options available for Brazil are the Wound-Qol(9,10) and the FLQA-Wk.(11) Both were developed in Germany, and translated and adapted for Brazil. The Wound-QoL covers 17 items assigned to three subscales: everyday life, body, and psyche. Besides assessing QoL, the Wound-QoL is easy to fill out and of brief application, responded by patients in a simple way, and giving professionals quick way to measure the construct. It has questions on a Likert scale, ranging from 0 to 4, where zero indicates the worse QoL and four the better QoL. The instrument is self-explanatory and must be completed by the patients, but they can receive help if they are not able to complete it on their own, what is documented if happens. Its validation had a Cronbach’s Alpha of 0.84 and a criterion validity concurrent with the FLQA-Wk of strong magnitude (0.85), considered a great result and confirming a reliable internal consistency, being satisfactory for the Brazilian culture.(9,10)

The Freiburg Life Quality Assessment-Wound (FLQA-w) scale consists of 24 items. It is a more complete scale, in a Likert format, with a score varying from one (best QoL) to five (worst QoL), with exception of the satisfaction domain. It presents six domains: physical symptoms, daily life, social life, psychological well-being, treatment and satisfaction. The validation study to Brazilian Portuguese showed good psychometric properties.(11)

A non-probabilistic sample of 100 patients with CW who were attending the institutions participated in the study, 92 from the HU and eight from the BHU. Patients were invited to participate in the research when they came for a dressing at the outpatient clinic and met the following inclusion criteria: age equal to or over 18 years, being in consultation for evaluation and dressing of one or more chronic wounds characterized as: wounds that did not heal in 3 months, or that did not show 20 to 40% of healed area after 2 to 4 weeks of treatment. Patients with altered cognitive and mental state reported in the medical records, who did not have conditions of communication to answer the questionnaires, or who had neoplastic CW were excluded (aiming to eliminate the bias of QoL alteration due to cancer diagnosis and symptoms of disease).

Data were analyzed using the Statistica software version 7.0. Sociodemographic and clinical data were descriptively analyzed by simple and absolute frequency. Wound-QoL and FLQA-Wk data were presented in domains with descriptive measures (mean, minimum, maximum and standard deviation) according to the manual scoring of both instruments. The Student t-test and Mann Whitney test were used for correlations between sex and the means of the Wound-QoL and FLQA-Wk domains. The Student t-test, Mann Whitney test, and ANOVA were used for correlations between schooling and means of the Wound-QoL and FLQA-Wk domains, and the Spearman test was used for the correlation of age with the means of the domains.

The research was approved by the Research Ethics Committee of the Federal University of Paraná on June 14, 2017, with Opinion nº 2,119,702 and by the Municipal Health Secretariat of Curitiba with the nº 88/2016.

## Results

The sample consisted of 100 patients, with an average age of 60.98 years and an age range ≥ 60 years to 66. Of the patients, 51 were male, 53 were married, 40 had more than 3 children, and 72 had elementary school. Regarding occupation, 75 were retired, and 55 received 1 to 3 minimum wages (Table 01).

**Table 1:** Sociodemographic characteristics of 100 patients with chronic wounds

Variables	*n*
Sex	
Male	51
Female	49
Age	
18 to 30 years	5
31 to 59 years	29
≥ 60 years	66
Marital status	
Married	53
Not married	13
Consensual Union	6
Separated	10
Widowed	18
Number of children	
0	11
1	10
2 to 3	39
More than 3	40
Schooling	
Incomplete Elementary School	66
Complete Elementary School	6
Incomplete high school	2
Complete high school	17
Complete high school technical course	2
Complete higher education	6
Incomplete higher education	1
Family income*	
Without income	6
Up to 1 minimum wage	30
1 to 3 minimum wages	55
4 to 10 minimum wages	7
10 to 20 minimum wages	2
Occupation	
Formal worker	4
Self-employed	10
Retired	75
Housewife	6
Unemployed	5

* 1 minimum wage in 2018: R $ 954 reais. Note: 1US dollar = R $ 3.88 reais.

Regarding clinical data, 41 patients had diabetic ulcers and 21 venous ulcers. As for the number of wounds, location and time of treatment, 65 had one wound, the predominant location was the lower limbs in 92 patients, and 83 treated the wounds for more than 24 months. The presence of comorbidities was predominant; 94 had some type of comorbidity, being Systemic Arterial Hypertension (SAH) and Diabetes Mellitus (DM) the most frequent. Medications were used by 88 patients, the most frequent of which were: antihypertensive, hypoglycemic/antidiabetic, anti-dyslipidemic/cardiovascular. Some patients used more than one medication (Table 2).

**Table 2:** Clinical characteristics of 100 patients with chronic wounds

Variables	*n*
Type of wound	
Diabetic ulcer	41
Venous ulcer	21
Calluses/ lesions leprosy	12
Osteomyelitis	7
Other wounds*	19
Nº of wounds	
1	65
2	21
≥ 3	14
Wound location	
Lower limbs (leg, thigh and foot)	92
Trunk (dorsal and ventral)	7
Lower limbs (leg / thigh) and	1
Wound Time	
6 to 18 months	14
19 to 23 months	3
≥ 24 months	83
Types of comorbidities †	
Hypertension	62
Diabetes	58
Dyslipidemia	17
Leprosy	14
Heart disease	14
Hypothyroidism	12
Stroke	5
Others	44
Medicines used †	
Antihypertensive drugs	63
Hypoglycemic/antidiabetic	51
Antidyslipidemic/cardiovascular	50
Antidepressants	16
Painkillers	13
Antibiotics	5
Others	44

(*): 3 cases of arterial ulcer, pressure injury, and livedoid vasculitis each, and one case of the following types: cellulitis, surgical ulcer, mycosis for diabetes, leishmaniasis ulcer, trauma, mixed, neurotic excoriation, bullous epidermolysis, suture dehiscence, arthritis and pressure injury; (†): Non-exclusive, the patient may use more than one option.

When analyzing the QoL of patients with CW with the Wound-Qol instrument, it was observed that the three domains had scores below the average, as well as the global value of 37.50, which denotes a low QoL. When analyzing the QoL with the FLQA-Wk instrument, similar results were found, the total score was below the average (44.20), which corresponds to a poor QoL. With the Wound-Qol, body symptoms were the ones with the lowest average, followed by symptoms of everyday life. When using the FLQA-Wk scale, the lowest averages were found for psychological well-being symptoms, followed by physical symptoms (Table 3).

**Table 3:** Quality of life scores measured with the Wound-Qol and FLQA-Wk in 100 patients with chronic wounds.

Wound-Qol	mean	SD	Min.	Max.
Body symptoms	21	20.50	0	80
Physical symptoms	46	30.25	0	100
Everyday life	34.75	27.50	0	100
Global	37.50	20.25	0	83.75
*FLQA-Wk*				
Physical symptoms	38	17	20	96
Daily life	51.20	21	20	100
Social life	43	23.40	20	100
Psychological well-being	34.20	17.80	20	10
Treatment	48.60	16.20	20	95
Satisfaction	50	18.20	20	93.40
Total	44.20	13	21.60	77.60

When comparing the body symptoms, physical symptoms and everyday life domains of the Wound-QoL and the physical symptoms, daily life, social life, psychological well-being, treatment domains of the FLQA-Wk, there were no significant differences between the variables sex, age and schooling. Age was correlated with the satisfaction item of the FLQA-Wk, with a *p*-value <0.001, verified with the Spearman’s correlation coefficient which obtained a value of 0.308, indicating that the higher the age, the lower the score in the satisfaction domain. It is worth mentioning that this item brings questions related to patients’ satisfaction about their general health, treatment and appearance of their wound. Its data were recoded to run the correlations, because they have an opposite presentation in relation to the other domains, that is, 1 indicates worst QoL and 5 best QoL.

## Discussion

When a patient has to live with a chronic wound, this health problem can cause changes and obstacles in several aspects in his life and that of his family members. They can be of a physical nature, when they prevent everyday life activities, or of an emotional nature, when they interfere with the patient’s way of living in the world. The presence of one or more CW, tend to lead the person to develop negative feelings, triggering a difficulty in interpersonal relationships, impairment of the body image and even of sexual activity, causing an impact that may harm the QoL.(4) Thus, it is up to health professionals to carry out humanized and holistic actions to develop a therapeutic itinerary aimed at the real needs of these patients.

The sociodemographic profile of the population of the present study showed the predominance of males, similar to results of studies carried out in other regions of Brazil,(9) and in Germany.(12) The experience of a patient with CW brings up aspects related to gender and health, such as sexuality and social implications, which trigger restrictions in daily life and in the performance of roles. These issues need to be considered by health professionals in order to improve the mental health care and QoL of these patients.(13)

Other important factors that must be considered for the preparation of the care plan of patients with CW are education and family income. In this study, the predominant level of education was elementary school. This is in line with studies carried out in the central west region of Brazil, where 77.36% of the patients had low schooling,(14) and in the validation study of the FLQA-Wk scale carried out in the southeastern region of Brazil, in which 97% had low schooling.(11) Regarding income, 55% of the patients received 1 to 3 minimum wages, higher than a study carried out in the northeastern region of Brazil, in which 85% of the elderly had low family income.(15)

Both low education and low family income have the potential to interfere in adherence to CW treatment. They can lead to an extension of recovery time and an impact on the patients’ QoL. Low schooling may influence the understanding of the guidelines on self-care.(8) Family income, for many patients, is primarily destined to cover the family needs such as food, clothing and education; when the materials and drugs prescribed for the wound are not provided in basic health units or specialty centers, adherence to treatment may be compromised.(16)

Regarding the data related to the QoL of patients with CW evaluated in this study with the instruments Wound-Qol and FLQA-Wk, it was observed that their averages were low in all domains, which denotes a poor QoL. Thus, the appearance of the wound has compromised the lives of the patients. The body symptoms were those that presented the worst average in the Wound-Qol. They are related to pain, odor, secretion of the wound, in addition to sleep and treatment. Similar data were found in a study carried out in Portugal which highlighted that the worsening QoL of patients with leg ulcers is related to pain, altered body image, bad odor, restricted mobility, exudate, negative emotions, sleep disorders, depression and anxiety.(17) These conditions demonstrate how relevant is that health professionals develop strategies related to clinical factors. The assessment of the lesion and the provision of appropriate treatment may reduce the impact of CW on these patients.(18)

Another domain compromised in the assessment of QoL was everyday life activities. They include difficulties in daily life such as climbing stairs, leisure activities and dependence on help from other people. In the FLQA-Wk, the lowest score was found for symptoms of psychological well-being, related to feelings towards the wound, such as: depression, tiredness and feeling of abandonment. Two studies carried out in the northeastern region of Brazil, one with the objective of evaluating the QoL of patients with venous ulcers(19) and the other with the objective of evaluating the QoL of patients with CW,(18) both using the Charing Cross Venous Ulcer Questionnaire, highlighted that the emotional and aesthetic domains had poor averages, associated with domestic activities and social interaction, and that the “well-being” domain had the lowest QoL score. Although the studies did not use a specific instrument to assess QoL, the results corroborate with the present study, detecting changes caused by CW in patients’ routine, and a consequent decline in QoL.

The presence of CW causes patients who were previously active to experience reduced work, social and daily life activities. The wound often requires patients to remain at rest for some periods of the day, so that these people start to isolate themselves and stop participating in their social cycles. Pain limits mobility and restricts domestic activities. The odor that can be exhaled can cause shame and lead family and friends to stay away.(4) These aspects show the extent to which the patients’ holistic assessment is pertinent and the extent of the impact on their QoL.

To survey the QoL of patients with CW and the costs involved were the objectives of a study conducted in Australia and Wales with data collected using the Cardiff Wound Impact Schedule (CWIS) questionnaire. The results showed that the global QoL and satisfaction scores were below ideal (60/100), physical symptoms and daily life scores were reasonable (64/100), and social life scores were higher (72/100), and well-being scores lower (40/100). Furthermore, younger participants reported worse QoL in all subscales of the CWIS when compared to older participants.(20) Another study conducted in Nigeria that assessed the QoL of patients with CW through from the World Health Organization-5 Well-being Index, 12-item General Health Questionnaire, 58% of the patients reported having adequate well-being and 41% were prone to depression and low mood. In addition, 32% suffered from anxiety and 15% from depression.(21)

Regarding the correlation of sociodemographic data with the domains of the Wound-QoL and FLQA-Wk, this research showed that there was no significant correlation between the domains and the variables education and sex. A study carried out in the central-western region of Brazil whose objective was to assess the QoL of patients with CW, using the WHOQOL-Bref questionnaire developed by the World Health Organization (WHO), did not show significant differences in relation to sex, concluding that this is a variable that does not influence the QoL of the evaluated patients. Another study carried out in the northeastern region of Brazil using the Ferrans & Powers Quality of Life Index - Wounded Version (IQVFP-VF), showed a correlation between the sex variable and the General QoL Index (*p*=0.04).(22)

In studies carried out in Brazil and in other places in the world, it is a consensus that QoL is related to the perception of each individual. therefore, it can be represented in different ways in each researched region. Perceptions of worse QoL may be related to worse living conditions, which do not favor health care, availability of sanitation, housing categories, low income, education and social relationships, as well as psychological problems. All of these aspects can compromise QoL in any abnormal health condition.

This study presented as a limitation a sample with visual difficulties, with difficulty to read, perhaps due to low education and income. A professional was available to assist in the reading of the instruments.

## Conclusion

It is concluded that the QoL of patients with CW assessed in this study using the instruments Wound-Qol and FLQA-Wk was poor. Both instruments showed scores below the average. The variables sex, age and education did not affect QoL. This indicates that multiprofessional teams need to develop actions aimed at individual as a whole, in order to provide an appropriate treatment and a possible improvement in the patients’ CW. However, the use of instruments for measurement of QoL in patients with chronic wounds is essential to assess, plan and structure a nursing care plan to optimize standardized procedures in the treatment of injuries.
